# Detection of shallow anterior chamber depth from two-dimensional anterior segment photographs using deep learning

**DOI:** 10.1186/s12886-021-02104-0

**Published:** 2021-09-22

**Authors:** Zhuyun Qian, Xiaoling Xie, Jianlong Yang, Hongfei Ye, Zhilei Wang, Jili Chen, Hui Liu, Jianheng Liang, Lihong Jiang, Ce Zheng, Xu Chen

**Affiliations:** 1 Department of Ophthalmology, Shanghai Aier Eye Hospital, No. 1286, Hongqiao Road, Changning District, Shanghai, 200050 China; 2 Aier School of Ophthalmology, Central South University Changsha, Changsha, Hunan Province China; 3 Joint Shantou International Eye Center of Shantou University and the Chinese University of Hong Kong, Shantou University Medical College, Shantou, Guangdong China; 4 Ningbo Institute of Industrial Technology, Chinese Academy of Sciences, Beijing, China; 5 Department of Ophthalmology, Xinhua Hospital, Shanghai Jiao Tong University School of Medicine, No.1665, Kongjiang Road, Yangpu District, Shanghai, 200092 China; 6 Department of Ophthalmology, Shanghai Children’s Hospital, Shanghai Jiao Tong University, Shanghai, China; 7 Department of Ophthalmology, Shibei Hospital, Shanghai, China; 8 Department of Ophthalmology, Zhabei Center Hospital, Shanghai, China; 9 Department of Ophthalmology, Shanghai Aier Qingliang Eye Hospital, Changning, China; 10 Aier Eye Hospital, Jinan University, No.601, Huangpu Road West, Guangzhou, P.R. China

**Keywords:** Deep learning, Anterior chamber depth, Anterior segment photographs

## Abstract

**Background:**

The purpose of this study was to implement and evaluate a deep learning (DL) approach for automatically detecting shallow anterior chamber depth (ACD) from two-dimensional (2D) overview anterior segment photographs.

**Methods:**

We trained a DL model using a dataset of anterior segment photographs collected from Shanghai Aier Eye Hospital from June 2018 to December 2019. A Pentacam HR system was used to capture a 2D overview eye image and measure the ACD. Shallow ACD was defined as ACD less than 2.4 mm. The DL model was evaluated by a five-fold cross-validation test in a hold-out testing dataset. We also evaluated the DL model by testing it against two glaucoma specialists. The performance of the DL model was calculated by metrics, including accuracy, sensitivity, specificity, and area under the receiver operating characteristic curve (AUC).

**Results:**

A total of 3753 photographs (1720 shallow AC and 2033 deep AC images) were assigned to the training dataset, and 1302 photographs (509 shallow AC and 793 deep AC images) were held out for two internal testing datasets. In detecting shallow ACD in the internal hold-out testing dataset, the DL model achieved an AUC of 0.86 (95% CI, 0.83–0.90) with 80% sensitivity and 79% specificity. In the same testing dataset, the DL model also achieved better performance than the two glaucoma specialists (accuracy of 80% vs. accuracy of 74 and 69%).

**Conclusions:**

We proposed a high-performing DL model to automatically detect shallow ACD from overview anterior segment photographs. Our DL model has potential applications in detecting and monitoring shallow ACD in the real world.

**Trial registration:**

http://clinicaltrials.gov, NCT04340635, retrospectively registered on 29 March 2020.

## Introduction

Anterior chamber depth (ACD) is an important biometry parameter for the diagnosis and therapy of ocular disease. In ophthalmology, AC depth measurements have several important applications, such as screening primary angle-closure glaucoma (PACG), calculating the power of intraocular lenses to be implanted after cataract extraction, and identifying the association with systemic parameters [1–3]. Shallow AC was proved to be associated with age, female gender, hyperopia, small optic disk, short body stature, and chronic angle-closure glaucoma [4]. Population screening for AC depth has been suggested to be useful in identifying subjects at risk of PACG [5]. Currently, the traditional methods for ACD measurement include slit-lamp biomicroscopy, IOLMaster, A-Scan ultrasound, and Pentacam [1, 6–8]. However all these techniques are time consuming and require trained and experienced technicians. A fully automated system can improve the accessibility of ACD measurement by creating a large-scale screening systems with overview eye images.

Deep learning (DL) models, such as deep convolutional neural networks, has become the state-of-the-art methodology for analyzing medical images, such as for the automated diagnosis of skin cancer, COVID-19, and glioma [9–11]. DL models have also been used across different sub-specialties in ophthalmology, such as in detecting diabetic retinopathy, classifying retinal disorders in optical coherence tomography images, and identifying age-related macular degeneration from fundus photographs [12–14]. However, to the best of our knowledge, no study has yet investigated the use of a DL model for the detection of an anterior segment image-based shallow ACD. Therefore, this study aimed to implement and evaluate a DL approach for detecting shallow ACD in anterior segment photographs.

## Methods

This study was approved by the Institutional Review Board of Shanghai Aier Eye Hospital (IRB: SHAIER2020IRB10) and conducted in accordance with the tenets of the Declaration of Helsinki, as revised in 2013. Informed consent was waived because of the retrospective nature of the fully anonymized images.

### Two-dimensional (2D) anterior segment imaging and anterior chamber (AC) depth measurement

In this study, we used a Pentacam HR system (Oculus Optikgerate GmbH, Wetzlar, Germany) to capture anterior segment photographs and measure the AC depth. The details of the Pentacam HR system have been described previously [15]. Briefly, the Pentacam is a high-resolution rotating Scheimpflug camera system for anterior segment (cornea, iris, and crystalline lens) imaging and analysis. It has one front infrared camera to capture the 2D overview eye image to evaluate the pupil size. It has been proven to be a noninvasive, repeatable, accurate, and reliable method for the measurement of anterior segment parameters, such as AC depth and corneal thickness [8, 16, 17]. After 5 min of dark adaption, the patients were asked to stare at a fixed light until a perfect alignment between the visual axial and the machine sensor was obtained. The Pentacam system then used a 360° rotating Scheimpflug camera with a monochromatic slit-light source (blue light-emitting diode at 475 nm) to calculate a three-dimensional (3D) anterior segment model and to capture photographs. The Pentacam software automatically calculated the AC depth, which is the distance from the corneal endothelium to the anterior surface of the lens, defined as the true ACD in Aung’s study [6]. Similar to Aung’s study, we also defined shallow AC as an AC depth of less than 2.4 mm. The anterior segment photographs taken by the front camera were saved anonymously in JPEG format for further analysis.

### Image datasets

The anterior segment photographs were selected from the cataract clinical databases of Shanghai Aier Eye Hospital from June 2018 to December 2019. All photographs were reviewed by licensed ophthalmologists. The exclusion criteria for image grading were [1] images without a quality parameter of the Pentacam HR marked “OK;” [2] corneal diseases such as scar, corneal degeneration, and pterygium; and [3] any sign of a previous eye surgery, such as corneal nebula after pterygium excision, pseudophakia, and filtering belb after trabeculectomy.

The whole image dataset was further randomized into training (90%) and testing datasets (10%). The training dataset was used to train the DL network, and the testing dataset was used to evaluate the algorithm. We used Tensorflow (https://www.tensorflow.org) to interpolate image pixels to fill a 299 × 299 matrix (tf.image. ResizeMethod with NEAREST_NEIGHBOR algorithm), with values in the range of 0–1 for the DL model training. Preprocessed training images were further augmented with Keras ImageDataGenerator (https://keras.io/) using various methods, including horizontal flipping, rotation, sharpening, adjustments to saturation, and zooming. We used k-fold cross-validation (k = 5) to train and evaluate the performance of the DL model [18]. This method has been commonly used in machine learning applications to avoid overfitting when dataset is small. In the cross-validation, the training dataset was split into k groups, with the (k − 1) groups used as the training data and one group for validation. The training process was repeated k times to allow for the use of all subsets exactly once as a validation dataset. During the training process, the DL model is trained on the training data and the validation data is used to tune the hyperparameters [19]. To evaluate the DL model, besides the first testing data collected from the cataract clinic, we further recruited the second testing data collected from glaucoma clinical dataset in the same center from March 2020 to December 2020.

### Development of the DL model

To detect shallow or deep AC from anterior segment images, we proposed the use of transfer learning based on the pre-trained Inception-V3 (Google, Inc.) architecture [20]. A pre-trained model is a saved network that was previously trained on a large-scale image classification task (ImageNet includes 1000 object categories with more than one million images) [21]. Therefore, this model effectively serves as a generic model of the visual world. We further fine-tuned the higher-order feature representations in the pre-trained Inception-V3 to make the model more relevant to our specific task. The subsequent DL architecture consisted of one GlobalAveragePooling layer, one hidden fully connected layer (including 256 neurons), and a final sigmoid classification layer to the output activation of shallow or deep AC. We used the rectified linear unit (ReLU) activation function to solve the vanishing gradient problem and the Adam optimizer (learning rate = 0.0001) with a minibatch size of 32 to update the weights and biases of the fine-tuned model. The model was trained for 100 epochs with the absence of further improvement in both accuracy and cross-entropy loss. The DL model was trained and validated using Keras API (version 2.2.4), with the Tensorflow framework (Google, version 2.1.0) as the backend. ﻿The computer hardware used had the following specifications: NVIDIA GTX 1080Ti 12 GB GPU (﻿NVIDIA, Santa Clara, CA, USA), 128 GB RAM, and Intel Core i7-2700K 4.6 GHz CPU (Intel, Santa Clara, CA, USA).

### Evaluation of the DL model for detecting a shallow AC from anterior segment photographs

We used t-distributed stochastic neighbor embedding (t-SNE) ﻿to visualize the high-dimensional features learned by the DL model in two dimensions [22]. ﻿In the t-SNE scatter plot, t-SNE converts the similarities between data points from the extracted hierarchical features (256 features from the current InceptionV3 model), and each point corresponds to an individual anterior segment image, with similar images appearing nearer to one another than dissimilar images.

**Fig. 1:**
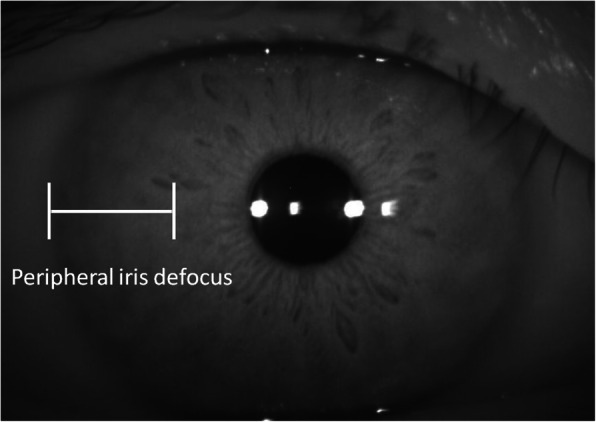
The AC was graded as shallow by the ophthalmologists if there has more than half length of the peripheral iris shadow or defocus in 2 dimensional anterior segment photographs

Using AC depth of less than 2.4 mm as the reference standard, a human-machine comparison was performed to evaluate the performance of the DL model in the internal hold-out testing dataset. Two ophthalmologists (L.JH and Q.ZY with 2 and 10 years of clinical experience, respectively), who were blinded from the dataset collection, were instructed to classify each image independently. We modified oblique flashlight beam methods to identify shallow AC. The AC would be graded as shallow if there has more than half length of the peripheral iris shadow or defocus in 2 dimensional anterior segment photographs (Fig. 1) [23]. The time for the evaluation of each image was controlled in 1 min.

To visualize the learning procedure of our DL model, we used the gradient-weighted class activation mapping (Grad-CAM) method to create heatmap images that indicated where the DL model was focused [24]. The Grad-CAM algorithm computed ﻿the weighted sum outputted by the last convolutional layer of the DL model. A heat map was then generated based on the grad-CAM to highlight the area for the DL model detection. The grad-CAM algorithm was coded using the Keras API and the Tensorflow framework as mentioned above. An experienced ophthalmologist (C.Z.) reviewed the photographs misclassified by the DL model and categorized them according to the two most commonly seen features: [1] images with coexisting eye conditions (e.g., ptosis, poor eye exposure, and tearing eye) and [2] images with coexisting photo conditions (e.g., incorrect exposure and off-center).

### Statistical analysis

We used confusion matrices to compare the prediction of DL models with the reference standard (ACD < 2.4 mm). The matrices included the area under the receiver operating characteristic curve (AUC) of the receiver operating characteristic (ROC) curves, accuracy, sensitivity, and specificity. The ROC curve was plotted by applying different thresholds to the output score maps from the DL model. The closer the AUC is to 1, the better the DL model. Accuracy, sensitivity, and specificity are expressed as follows:

1 $$ \mathrm{Accuracy}=\frac{TP+ TN}{TP+ TN+ FN+ FP,} $$

2 $$ \mathrm{Sensitivity}=\frac{TP}{TP+ FN}, $$

3 $$ \mathrm{Specificity}=\frac{TN}{TN+ FP}, $$

TP, TN, FP, and FN represent true positive, true negative, false positive, and false negative, respectively. Python (version 3.7) and Scikit_learn modules (Anaconda, version 1.9.12, Continuum Analytics) were used to perform the statistical analysis.

## Results

**Fig. 2:**
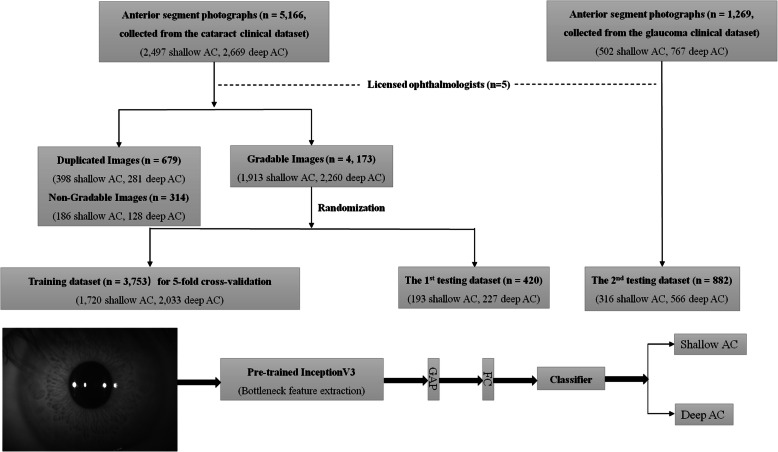
Flowchart of the grading and randomization processes of the image datasets

The whole raw dataset consisted of 5166 anterior segment photographs with 2497 shallow AC (AC depth < 2.4 mm) and 2669 deep AC (AC depth ≥ 2.4 mm) images from 4562 subjects (1786 males and 2776 females with a mean age of 54 ± 9 years). To prevent the data correlation, we only selected one image for each subject (excluding 679 images with 398 shallow AC and 281 deep AC respectively). A total of 314 images (﻿186 shallow AC and 128 deep AC images) were further excluded by five licensed ophthalmologists because of poor image quality, leaving behind 4173 images (from 4173 subjects) with AC depth measurement (Fig. 2). Using a simple random sampling method, 3753 images (﻿1720 shallow AC and 2033 deep AC images) were assigned to the training dataset, and the remaining 420 images (193 shallow AC and 227 deep AC images) were held out for the first internal testing dataset. The second raw testing dataset included 1269 AC images (502 shallow AC and 767 deep AC images). 387 images were excluded as images were also recorded in training dataset, leaving behind 882 AC images (316 shallow AC and 566 deep AC images). For the second testing dataset, we only enrolled one image for each subject.

**Fig. 3:**
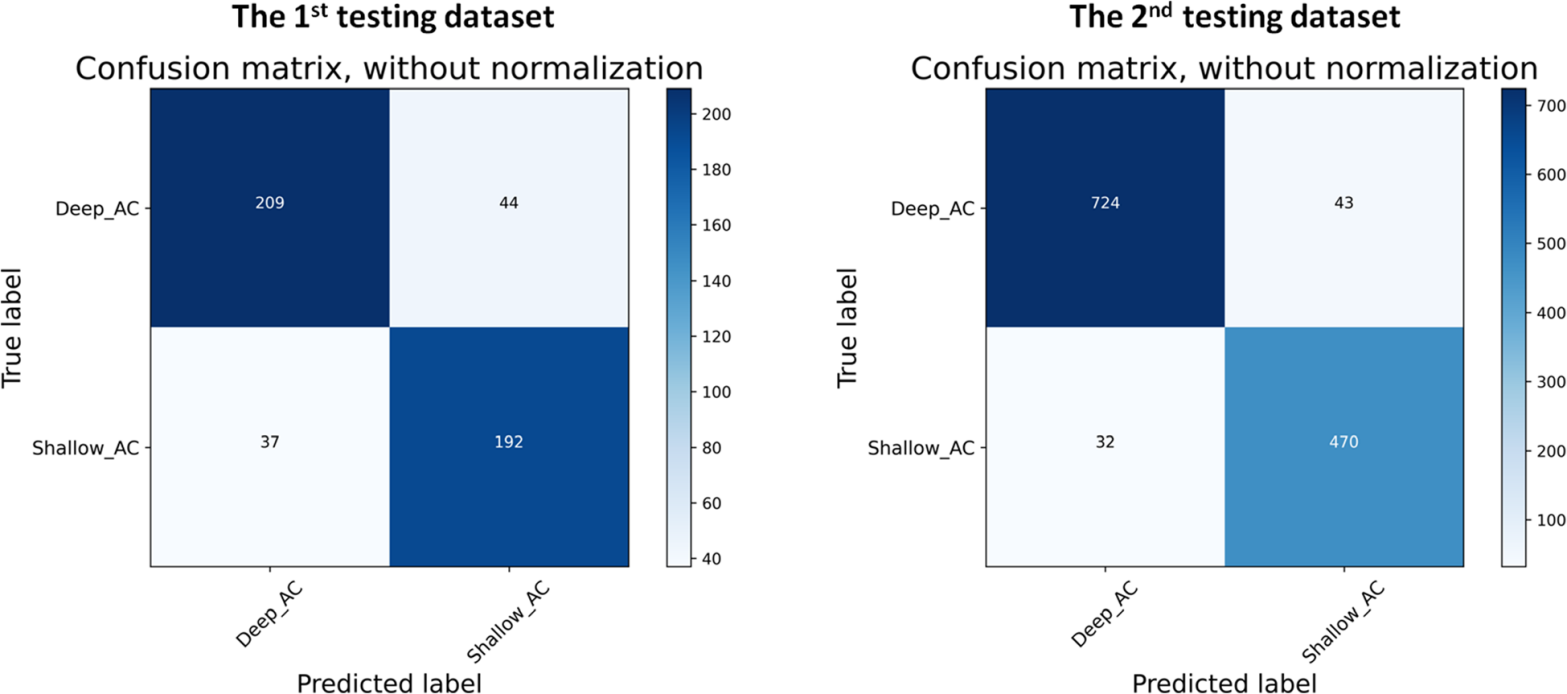
Confusion matrix for the DL model testing in two internal testing datasets

﻿In the first testing dataset, the AUCs, sensitivity, specificity, and accuracy of the proposed DL model for shallow AC (AC depth < 2.4 mm) detection were 0.86 (95% CI, 0.83–0.90), 0.80 (95% CI, 0.76–0.84), 0.79 (95% CI, 0.75–0.83), and 0.80 (95% CI, 0.76–0.84), respectively (Table 1 and Fig. 3). Compared with the diagnostic performance of the DL model, two ophthalmologists showed limited ability to identify shallow AC from anterior segment photographs, with an accuracy of 0.74 (95% CI: 0.70–0.78) and 0.69 (95% CI: 0.65–0.73), respectively. In the second testing dataset, the AUCs, sensitivity, specificity, and accuracy of the proposed DL model for shallow AC detection were 0.93 (95% CI, 0.91–0.95), 0.85 (95% CI, 0.83–0.87), 0.90 (95% CI, 0.88–0.92), and 0.88 (95% CI, 0.86–0.90), respectively (Fig. 3).

**Table 1:** The diagnostic performance of DL_Model and human graders testing in internal hold-out testing dataset

	Accuracy (95% CI)	Specificity (95% CI)	Sensitivity (95% CI)
Deep learning models	0.80 (0.76 to 0.84)	0.79 (0.75 to 0.83)	0.80 (0.76 to 0.84)
Human experts			
#1 Glaucoma specialist	0.74 (0.70 to 0.78)	0.81 (0.78 to 0.85)	0.68 (0.64 to 0.72)
#2 Glaucoma specialist	0.69 (0.65 to 0.73)	0.65 (0.61 to 0.69)	0.72 (0.68 to 0.76)

**Fig. 4:**
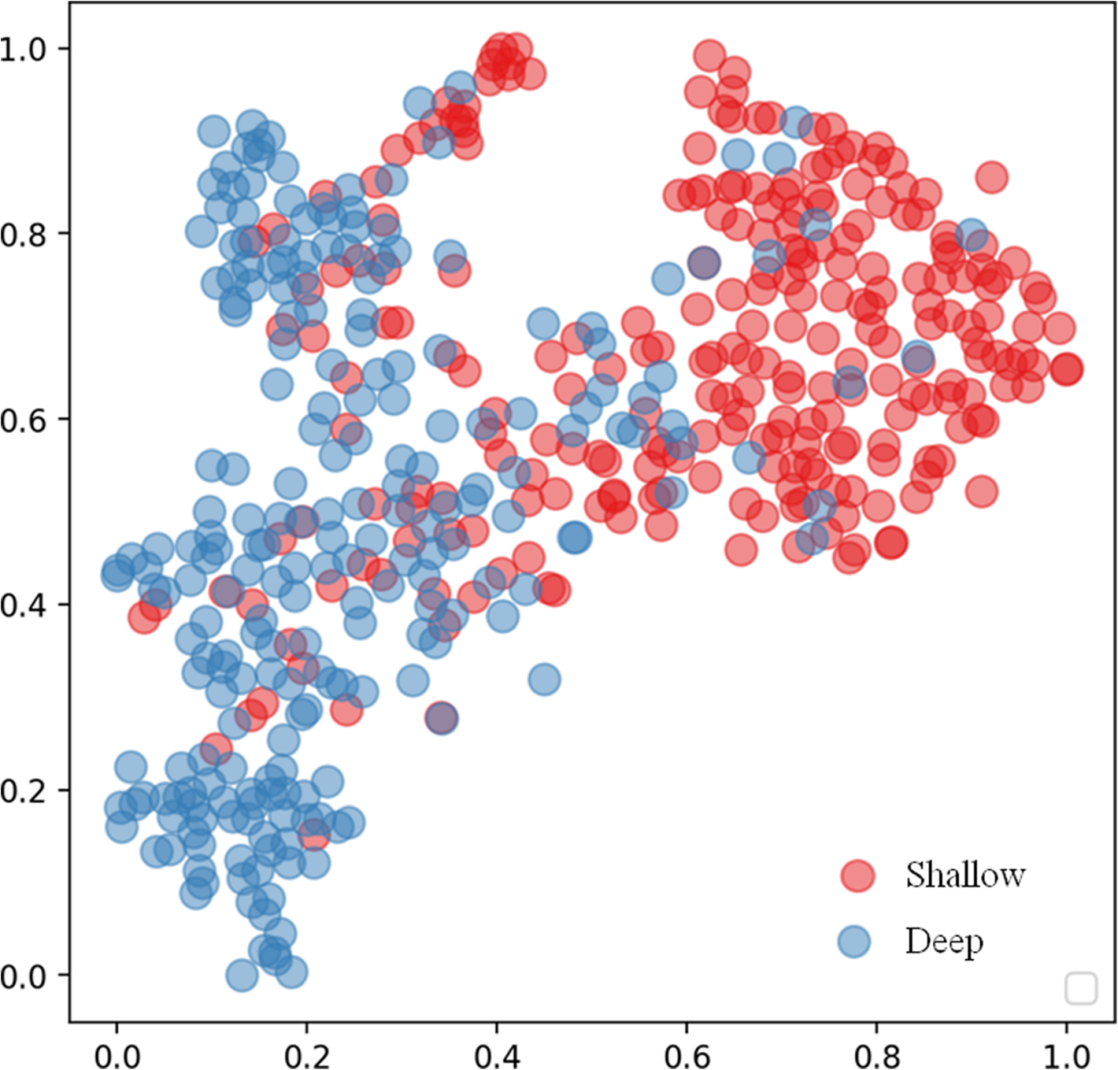
t-distributed stochastic neighbor embedding visualization of the features extracted from a fully connected layer of the DL model for shallow AC detection using AC depth of less than 2.4 mm as the reference standard

Figure 4 shows the t-SNE visualization of the ﻿high-dimensional features learned by the DL model in two dimensions. This visualization demonstrated that the DL model was able to automatically generate features that roughly detect shallow AC from anterior segment photographs using an AC depth of less than 2.4 mm as the reference standard.

**Fig. 5:**
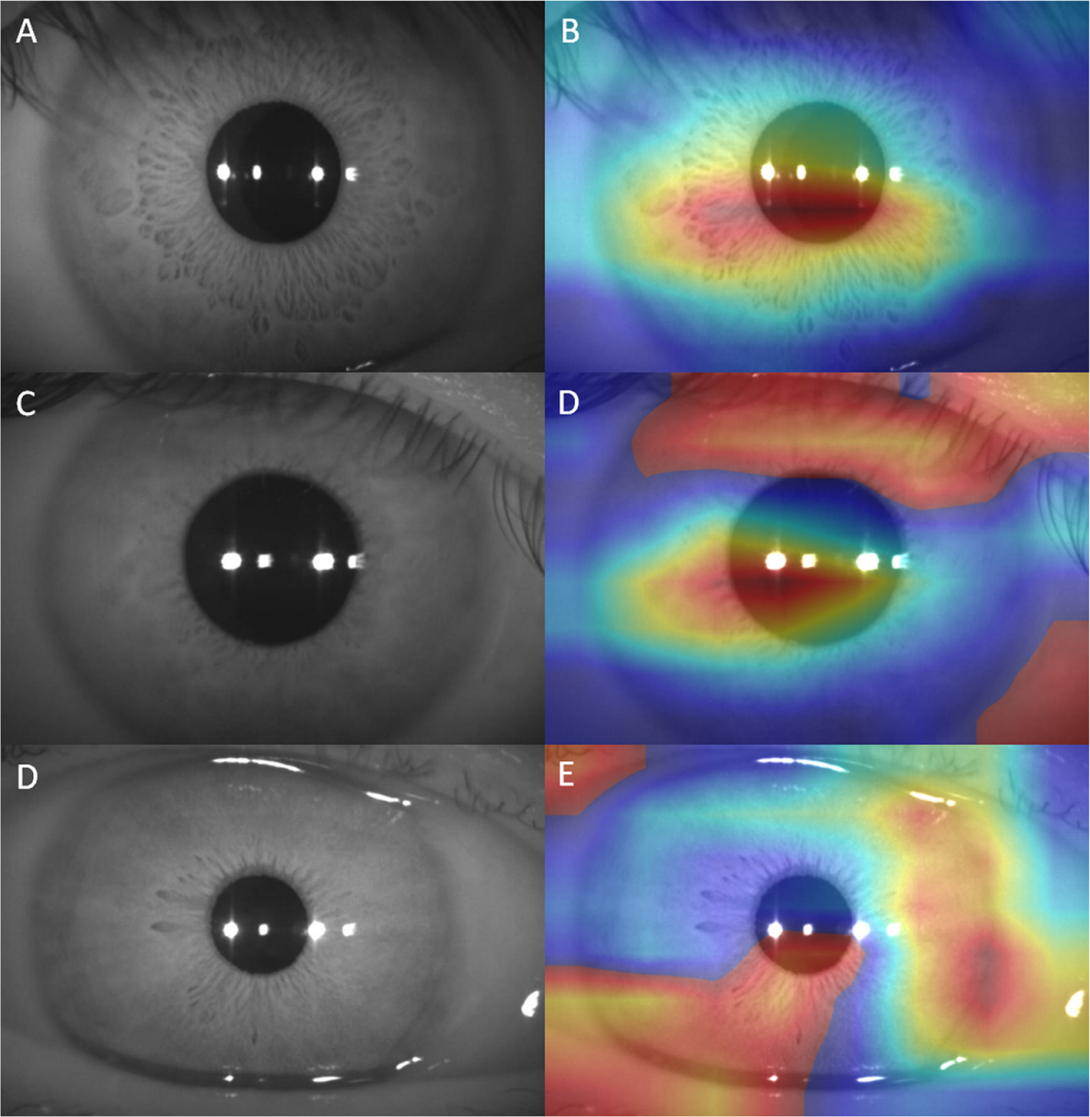
Anterior segment photographs showing correct and false classification cases. A, B: A deep AC image with AC depth = 3.12 mm and Grad CAM highlighting the central AC, cornea, and surrounding iris area. C, D: A shallow AC image (AC depth = 2.07 mm) was misclassified by the DL model, and the image was defocused on the eyelid highlighted by Grad CAM. E, F: A deep AC image (AC depth = 2.96 mm) was misclassified by the DL model. The image shows a high lacrimal meniscus, which is highlighted by Grad CAM

Figure 5A–B show a Grad CAM generated from the InceptionV3 model. Interestingly, activation was mostly shown in the central AC and the surrounding iris area. Table 2 shows the proportion of the reasons for misclassification by the DL Model in the internal hold-out testing datasets. The most common reason for misclassification was the images had coexisting photo conditions (*n* = 57, 70.4%). Figure 5C–D shows a shallow (AC depth = 2.07 mm) AC image misclassified by the DL model. The photo was defocused on the eyelid, which was highlighted by Grad CAM. Another reason for misclassification was the images had coexisting eye conditions (*n* = 24, 29.6%). In Fig. 5E–F, a deep AC image (AC depth = 2.96 mm) was misclassified by the DL model. The image shows a high lacrimal meniscus, which was also highlighted by Grad_CAM.

**Table 2:** The proportion of reasons for misclassification by the deep learning model in internal hold-out testing datasets

Reason	No. (%)
With coexisting eye conditions	24 (29.6%)
With coexisting photo conditions	57 (70.4%)

## Discussion

In this study, using the pre-trained Inception-V3 with transfer learning, the proposed DL model showed the capability of automatically detecting shallow or deep AC directly from overview anterior segment photographs without slit-lamp illumination. A comparison of the diagnostic accuracy between ophthalmologists and the DL model revealed that ophthalmologists were less likely to detect shallow AC through the anterior segment appearance. To the best of our knowledge, this study is the first to report the classification ability of a DL model with high accuracy in shallow AC detection using anterior segment photographs.

In the clinical works, several clinical techniques have been proposed for AC depth measurements, such as IOLMaster, A-Scan ultrasound, and Pentacam [1, 6–8]. However, these techniques are expensive and require trained nurses or technicians. In the current study, the DL model requires only photos and shows a higher accuracy (0.83 with 95% CI, 0.80–0.86) in screening shallow AC (AC depth < 2.4 mm) in the clinical hold-out dataset. Most medical DL systems ﻿adopt senior doctors’ grading as ground truth, but this grading system ﻿is time consuming and inherently subjective. ﻿The proposed DL model used the quantitative measurement of Pentacam as the gold standard to grade the dataset, and it made the results more objective and reliable. Such advantages make the DL technique an efficient means of screening the general population.

AC depth is a 3D biometric parameter associated with the ﻿anatomical structures of the anterior segment, such as the lens vault and the posterior corneal arc length [25]. In clinical practice, ophthalmologists can qualitatively assess the AC using the pen torch method, the slit-lamp van Herick technique [26], or the Smith method [27]. As knowledge and clinical experience vary among different individuals, human performance shows large variations in these techniques. Moreover, it is difficult to detect shallow AC directly from anterior segment photographs because of the limitation of 3D information. DL may address this by learning the critical features from a high-dimensional space [28, 29]. For classification tasks, higher layers of the DL model amplify the aspects of the input that are important for the discrimination and suppression of irrelevant variations. Varadarajan [29] successfully used DL to make predictions using simple 2D images by fundus photography without sophisticated 3D imaging equipment in diabetic macular edema grading. In our study, we used t-SNE to create a 2D reduced representation of the 256-dimensional space extracted from the last fully connected layer of the DL model (Fig. 4). Our result shows that the DL model is able to automatically generate features that roughly detect shallow AC from anterior segment photographs using AC depth of less than 2.4 mm as a reference standard.

Grad CAM is an algorithm used to create heatmap images that indicate where the DL model is focused. Note that Grad CAM highlights the central AC, cornea, and surrounding iris area, which is also where ophthalmologists assess the AC during routine clinical practice [27]. Grad CAM may also uncover the reasons that cause false predictions of the DL model [30]. In the current study, the most common reason for misclassification is the images with coexisting photo conditions, especially those that were defocused during photography. Figure 5C and D show that the DL model highlights the eyelid area, which was focused when the photograph was taken. This issue can be solved using an advanced imaging technique, such as auto-focus [31]. Another reason for misclassification is the images with coexisting eye conditions, such as high lacrimal meniscus (Fig. 5E–F). These nontraditional highlighted regions may offer some additional information for ﻿further investigation by eye care professionals.

﻿This study has several limitations. First, the Pentacam camera uses a monochromatic slit-light source to produce only black and white images. Fortunately, the DL model can be adopted to train with color images with other imaging modalities. Second, the sample size of the training dataset is relatively small, and the model can only predict shallow or deep AC, not a specific value of AC depth. ﻿Third, all the subjects involved in the study were Chinese. Future studies with more subjects of multiple ethnicities and multiple imaging modalities, such as mobile phone eye photography, will be beneficial to provide more general predictions for clinical practice or community screening. Fourth, In the current study, DL model achieved better performance in the 2nd testing dataset (collected from glaucoma clinical dataset) than that in the 1st testing dataset (collected from cataract clinical dataset). We presumed that different AC depth between two testing datasets may affect the performance of DL model. The AC depth of shallow AC subjects in the 2nd dataset was shallower than that in the 1st dataset (1.84 ± 0.09 mm vs. 2.05 ± 0.13 mm, with *p* < 0.01). Our DL model cannot directly predict AC depth which may be more useful in clinical practice. We are developing another DL model to predict AC depth or volume using more data and will report our results in the future.

In conclusion, we proposed a DL model that can automatically detect a shallow AC based on anterior segment photographs. The results suggest that this DL model may be a potential tool for routine eye screening. Future efforts involving multiple ethnicities and multiple imaging modalities are warranted to investigate the application of this technology in the clinical and research setting or in community screening.

## Data Availability

The datasets used and/or analysed during the current study available from the corresponding author on reasonable request.

## Abbreviations

ACD: anterior chamber depth
PACG: primary angle-closure glaucoma
DL: deep learning
2D: two-dimensional
3D: three-dimensional
AC: anterior chamber
